# Inclusive LGBTQIA+ healthcare: An interprofessional case-based experience for cultural competency awareness

**DOI:** 10.3389/fpubh.2022.993461

**Published:** 2023-01-04

**Authors:** Samiksha Prasad, Chasity B. O'Malley, Rolando DeLeon, Arkene S. Levy, Daniel P. Griffin

**Affiliations:** ^1^Dr. Kiran C. Patel College of Allopathic Medicine, Department of Medical Education, Nova Southeastern University, Fort Lauderdale, FL, United States; ^2^Boonshoft School of Medicine, Wright State University, Dayton, OH, United States; ^3^Division of Obstetrics and Gynecology, HCA Florida Mercy Hospital, Miami, FL, United States

**Keywords:** cultural competency, interprofessional, inclusivity, Case-Based Learning (CBL), active-learning, gender minorities, LGBTQIA+ health

## Abstract

**Introduction:**

Lesbian, Gay, Bi-sexual, Transgender, Questioning, Intersex, and Asexual (LGBTQIA+) patients report experiences of discrimination within healthcare settings due to a lack of provider knowledge and biases of healthcare workers. There is an identified need among all health professions to provide more culturally competent healthcare for this community. Early interventions during healthcare profession training programs may be effective to address this need. The overall goal of this study was to assess the educational impact of an active learning session that was specifically designed to enhance LGBTQIA+ cultural competency awareness using an interprofessional setting.

**Methods:**

This 2-year study involved students from 16 healthcare professional programs joining virtually to form interprofessional teams. A small group case-based learning approach was used and included pre/post-activity surveys to measure the change in student attitude and confidence, as well as the change in perception of the importance of the activity.

**Results:**

Results indicate an increase in perception of importance (*p* < 0.005) and in overall level of confidence (*p* < 0.001) with respect to LGBTQIA+ issues post-session. Key themes established through the session represent an overall recognition of the importance of interprofessional education and awareness of LGBTQIA+ healthcare needs.

**Discussion:**

The results demonstrate the effectiveness of a case-based approach for enhancing cultural competency awareness across different healthcare professions programs. This session also provided an interprofessional learning environment to allow multiple healthcare professions program students to interact and share perspectives. The positive impact of this intervention in a highly collaborative virtual learning environment also highlights that this immersive active learning approach that can be adopted across different programs and institutions.

## Introduction

When seeking healthcare, Lesbian, Gay, Bi-sexual, Transgender, Questioning, Intersex, Asexual (LGBTQIA+) people are impacted by significant barriers. These barriers can take the form of disrespectful attitudes, discriminatory treatment, inadequate understanding of needs, and inability to provide appropriate care (1, 2). According to the 2019 southern LGBTQIA+ health survey conducted by the Campaign for Southern Equality, out of the 5,617 LGBTQIA+ patients who participated, it was reported that they delayed seeking healthcare because of their LGBTQIA+ identity and fear of discrimination from health care providers (3). LGBTQIA+ patients also reported alarmingly higher rates of suicidal ideation, depression, and anxiety than the general population, with the rates particularly high for transgender participants (3). The national transgender discrimination survey revealed that 19% of transgender persons are denied care based on their gender status, and 28% postponed care due to perceived harassment within a healthcare setting (4, 5). Intersex populations have limited research focused on their healthcare, but there is a tendency for intersexual adults to avoid healthcare due to traumatic healthcare experiences during childhood (6, 7). These disparities in physical health and quality of care for LGBTQIA+ patients highlight the need to improve their healthcare experiences by providing focused training to healthcare professional students (8).

It is important to provide training to healthcare professional students on the ways LGBTQIA+ people may experience barriers in healthcare settings to give students the tools they need to actively engage in reducing and eliminating these healthcare disparities for their future patients (1). Proficiency training of healthcare personnel and students has been shown to mitigate biases, discrimination, and microaggressions in learning environments by increasing the knowledge and cultural awareness of the faculty, staff, and students (9). Such trainings improve cultural awareness and proficiency; and translate to improved healthcare outcomes for the LGBTQIA+ population (10).

Providing comprehensive patient care requires collaborations between the various providers from multiple professions and specialties in healthcare organizations. This team-based approach within the organizations may have an underlying culture of care, that may be advantageous or detrimental to the patient, depending on the situation and those involved. For example, factors such as miscommunication between healthcare professionals could lead to an increase in hospital patients with at least one healthcare-associated infection according to the data from the Centers for Disease Control (CDC) (11). Interprofessional education (IPE) can help those in healthcare to not only better understand the existing organizational culture, but also apply changes to the culture of care to improve the care of their patients and health outcomes (12, 13). To do so, it is important to recognize and understand the distinguishing and mutual goals of individual professional groups caring for our patients. Through that understanding, we can develop solutions that allow for interprofessional education to help enhance collaboration and improve patient care. Establishing an understanding of one another's role in patient care and ways to work together has the potential to reduce error and improve the quality for care of our patients (14).

The Interprofessional Education Collaborative (IPEC), in 2016, updated the core competencies into a single domain of interprofessional collaborative practice with four sub-competencies: (i) values/ethics for interprofessional practice, (ii) roles/responsibilities, (iii) interprofessional communication, and (iv) teams and teamwork (15, 16). These competencies were emphasized for developing the case-based sequential disclosure active session. In accordance with the IPEC guidelines, Nova Southeastern University (NSU) Health Professions Division (HPD) holds an annual IPE Day. Due to the COVID-19 pandemic, NSU held the 2021 and 2022 IPE Day events virtually *via* the Zoom meeting platform. This enabled intercampus collaboration across eight campuses which included more than thirteen hundred (>1,300) students from eight (9) HPD colleges encompassing eighteen (17) professional programs.

In order to promote interprofessional (IP) communication between learners from different healthcare professional programs, an active learning approach is effective. Active learning is a student-centered concept denoting a participative process of engagement in classes and materials where students are involved in constructing their own learning (18, 19). For this intervention, Case-Based Learning (CBL) was determined to be the most appropriate method of delivery. CBL, through its various delivery methods, is used worldwide by many different fields and disciplines. CBL is defined in multiple ways in the literature, since it does not have a formal design, but instead will incorporate a variety of strategies based on the unique needs of the session (17).

With the oversight of facilitator(s) and stated learning objectives, CBL is structured to promote inquiry learning experience which includes patient cases to solve a clinically relevant problem (17). It is important to note that an advantage of CBL is that there is flexibility in its use depending upon multiple factors, such as the presence of pre-work, size of the group, number of facilitators, etc. CBL remains a methodology that is malleable and adaptable which may vary by institution and specific needs of the intervention. For this experience, pre-work was not feasible, therefore, information was given during the session and not prior as is common in the delivery of standard Problem-Based Learning (PBL) format (17).

This experience was designed for an I*P-*CBL, small group discussion with the primary goal of encouraging communication between healthcare professionals to help build an environment of inclusivity and support. Sessions such as this are at risk of having a diminishing impact unless additional sessions of this nature are added to ensure applicable skills are reinforced longitudinally throughout their professional training. Due to its malleability, this student-driven approach could be adapted by other schools and health professions programs to promote a comprehensive learning experience.

## Methods

### Educational objectives covered in the session

1. Demonstrate being receptive to the opinions of members of an interprofessional team in a patient-centered fashion. (IPEC domain fulfilled: Communications).

2. Discuss and clarify each profession's scope of practice and the roles of each healthcare professions team member. (IPEC domains fulfilled: Roles/Responsibilities and Communications).

3. Communicate the importance of teamwork in providing unbiased and inclusive patient-centered care. (IPEC domains fulfilled: Teams/Teamwork and Ethics).

4. Recognize boundaries experienced by a marginalized patient population (IPEC domains fulfilled: Ethics).

### Participants

One hundred and eighty healthcare professions students from 16 healthcare professional programs and 7 colleges participated in this virtual session for the IPE Day (2021–22) out of which 111 (61.67%) completed the pre/post-activity surveys for this study.

### Session context and logistics

#### Context

The 1-hour case-based sessions were held during IPE day in 2021 and 2022, respectively. This annual event is designed to introduce interprofessional concepts to students in the various health professions programs. The clinical vignette was designed to depict a bi-sexual female patient's experience during a visit to the doctor's office and subsequent experiences with other clinicians (Complete case in Appendix 1_Case). This allowed students to discuss the patient's experience from the perspective of the different health professionals involved.

#### Logistics

The sessions were hosted *via* the Zoom Meeting platform and repeated three times each year for a total of six sessions. For each session, students were randomly assigned into groups of 30 members each. The activity began with brief introductions and students were provided with the details for informed consent for the study. The anonymous pre-activity survey was then distributed using Microsoft Forms, accessible by hyperlink and QR code. The clinical vignette was revealed to students using sequential disclosure, through PowerPoint. Each part was read by a student member of the group. After each part, prompt questions were provided for group discussion. Clinical and basic science faculty were overseeing the group discussions and facilitated as needed. At the conclusion of the final discussion, the anonymous post-activity survey was disseminated.

### Data instrument

The anonymous pre-and post-activity surveys used a five-point Likert scale for obtaining the data. The pre-and post-activity surveys were not linked for individual participant responses to ensure student anonymity. Surveys were created based on revisions of the Health Disparities Attitudes and Knowledge Scale by Gavzy et al. (20) and Parker et al. (21). Human subjects research approval was obtained from the Nova Southeastern University Institutional Review Board for the pre-/post-activity surveys (IRB# 2021-12-NSU). The data instrument is provided as Appendix 2_Data Instrument.

### Data analysis

Each data category in the Likert scale was assigned the following numerical value for statistical analysis: Extremely Important/ Very Confident = 5; Somewhat Important/ Confident = 4; Neutral = 3; Somewhat Unimportant/ Minimally Confident = 2; Extremely Unimportant/ Not Confident at all = 1. Data was analyzed using GraphPad Prism Version: 9.3.1 (471). The data was aggregated, and an unpaired student *t-*test was used for analysis (a *p-*value of <0.05 was considered significant).

Demographics data was categorized into 5 different categories namely: (i) Health Professional College, (ii) Health Professional Program, (iii) Year of Study, (iv) Age Range, and (v) Gender. A prompt was included in the data instrument for any training received within the respective program curriculums prior to this experience. An independent samples *t-*test analysis was performed, to examine the significance of year of study and the number of hours of prior training.

Each individual narrative response was reviewed and tallied. Common themes were words/phrases appearing more than two times. The frequency of input of each common theme was used to plot an occurrence diagram.

## Results

### Demographics

Out of the 111 healthcare professional students participating in this study, 70.3% reported as female and 29.7% reported as male (Table 1). The age ranges of the participants included 63.1% comprising of 20–25 years old, 27.9% being in the 26–30-year age range, 5.4% being in the 31–35 years and 3.6% comprising 36–40-year age range (Table 1). The participant pool comprised primarily of students in their 1st year (44.2%) and 2^nd^ year (42.3%) of the study. Representation from the third year and fourth year of study was 11.7 and 1.8% respectively (Table 1). The results obtained from the independent samples *t-*test analysis for the effect on participant responses based on the year of study was not significant (all *p-*values obtained were >0.05).

**Table 1:** Participant demographics distribution.

	**Demographic** ** Category**	**Participant** ** distributions**	**Percent (%)**
1.	Participating Healthcare College	Healthcare Sciences	38.5
		Osteopathic Medicine	27.5
		Pharmacy	12.5
		Optometry	7.2
		Nursing	6.3
		Allopathic Medicine	5.4
		Dental Medicine	2.6
2.	Healthcare Program	Doctor of Osteopathic Medicine (DO)	24.4
		Physician Assistant (PA)	20.7
		Doctor in Pharmacy (PharmD)	12.6
		Doctor in Optometry (OD)	7.2
		Nursing (BSN)	6.3
		Doctor of Occupational Therapy (OTD)	4.5
		Anesthesiologist Assistant (AA)	4.5
		Doctor of Physical Therapy (DPT)	4.5
		Doctor of Medicine (MD)	2.7
		Doctor of Dental Medicine (DMD)	2.7
		Masters in Biomedical Sciences (MBS)	2.7
		Certificate of Health Professions (CHPP)	2.7
		Medical Sonography (DMS)	1.8
		Speech and Language Pathology (MS-SLP)	0.9
		Respiratory Therapy (RT)	0.9
		Registered Dietician (RD)	0.9
3.	Year of Study	1st year	44.2
		2nd year	42.3
		3rd year	11.7
		4th year	1.8
4.	Age Range (yrs.)	20–25	63.1
		26–30	27.9
		31–35	5.4
		36–40	3.6
5.	Gender	Female	70.3
		Male	29.7
		Other	0

Out of the participating 16 healthcare programs the top three belonged to the Doctor of Osteopathic Medicine (24.4%), Physician Assistant (20.7%), and Doctor in Pharmacy (12.6%) (Table 1). The complete breakdown of all participating programs is mentioned in Table 1. The participating students came from 7 Healthcare Colleges. The maximum representation was from the College of Healthcare Sciences (38.5%), College of Osteopathic Medicine (27.5%), and College of Pharmacy (12.5%) (Table 1).

The complete breakdown of all participants (*n* = 111) is grouped into 5 demographic categories (1: Participating Healthcare College, 2: Healthcare Program, 3: Year of Study, 4: Age Range, and 5: Gender).

### Prior training in LGBTQIA+ healthcare

**Figure 1:**
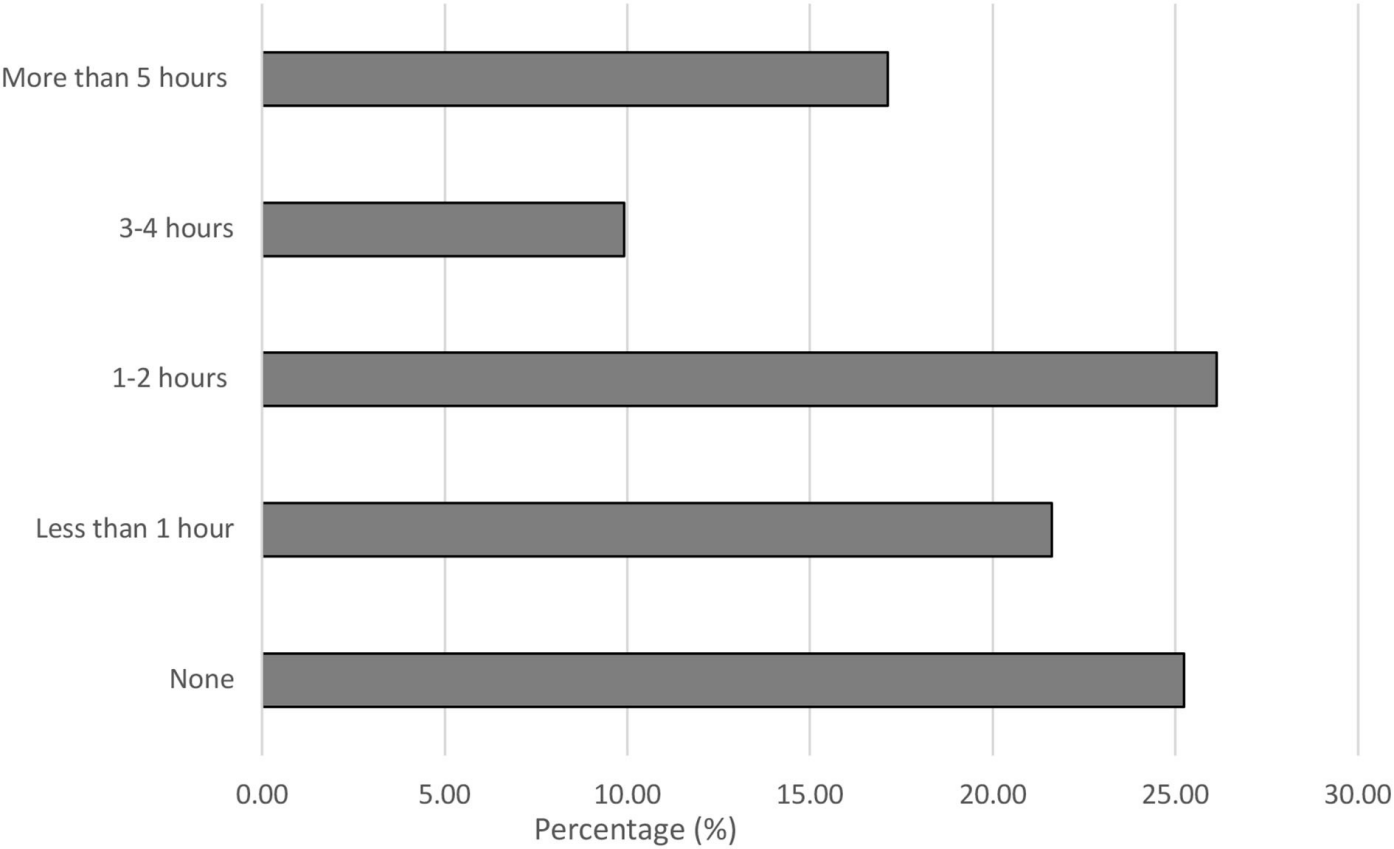
Reported prior training received in LGBTQIA+ healthcare. Participants (*n* = 111) responded to the following Prompt: (P0) How much prior teaching have you received in your current program exclusive to the care of lesbian, gay, bisexual, and transgender (LGBTQIA+) patients? Data is represented as a percentage of the total responses received.

Out of the 111 participants, 25.23% had no prior training exclusive to LGBTQIA+ healthcare. 21.62% of participants received <1 h training in the program curriculum. 26.13% had 1–2 h dedicated to LGBTQIA+ healthcare training. 9.91% of participants completed 3–4 h of prior training whereas only 17.12% had more than 5 h of dedicated training received in their current program exclusive to the care of LGBTQIA+ patients (Figure 1). The results obtained from the independent samples *t-*test analysis for the effect on participant responses based on the number of hours of prior training was not significant (all *p-*values obtained were >0.05).

### Change in importance

**Figure 2:**
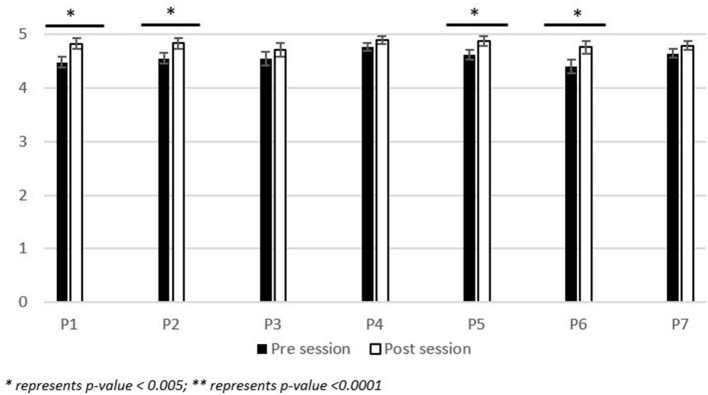
Participant reported change in perception of the importance of LGBTQIA+ topics. Range of significance of results of the pre-/post- activity survey responses (*n* = 111) to the following prompts: (P1) How important is it for healthcare professional students to receive education about the primary care of Lesbian, Gay, Bisexual, and Transgender patients? (P2) How important is it for healthcare professional students to receive education about the primary care of Transgender patients? (P3) How important is it for a primary care provider to be able to provide information to (LGBTQIA+) patients about local resources for (LGBTQIA+) community engagement? (P4) How important is it for healthcare professional students to recognize increased health risks associated with sexual orientation? (P5) How important is it to engage in self-reflection processes to correct implicit biases regarding LGBTQIA+ individuals? (P6) How important is it to implement gender-neutral practices in your clinical practice and clinic? (P7) How important is it to discuss safe sex practices with individual women who have sex with women? Participants responded on a 5-point Likert scale with 5, Extremely Important; 4, Somewhat Important; 3, Neutral; 2, Somewhat Unimportant; 1, Extremely Unimportant. Data is represented as the average response from the Likert scale +/– the standard error of the mean.

Prompts 1 through 7 (P1 through P7) capture the students' perspectives on the importance of questions related to LGBTQIA+ topics. Data is represented as a mean (Pre-activity vs. Post-activity data) +/– the standard error of the mean with a *p-*value of <0.05 considered as significant. From prompts P1 through P7, prompts P1 (4.49 vs. 4.83; +/– 0.10), P2 (4.56 vs. 4.84; +/– 0.10), P5 (4.62 vs. 4.88; +/– 0.09) and P6 (4.41 vs. 4.77; +/– 0.12) were significant (*p-*value <0.005) (Figure 2).

### Change in confidence

**Figure 3:**
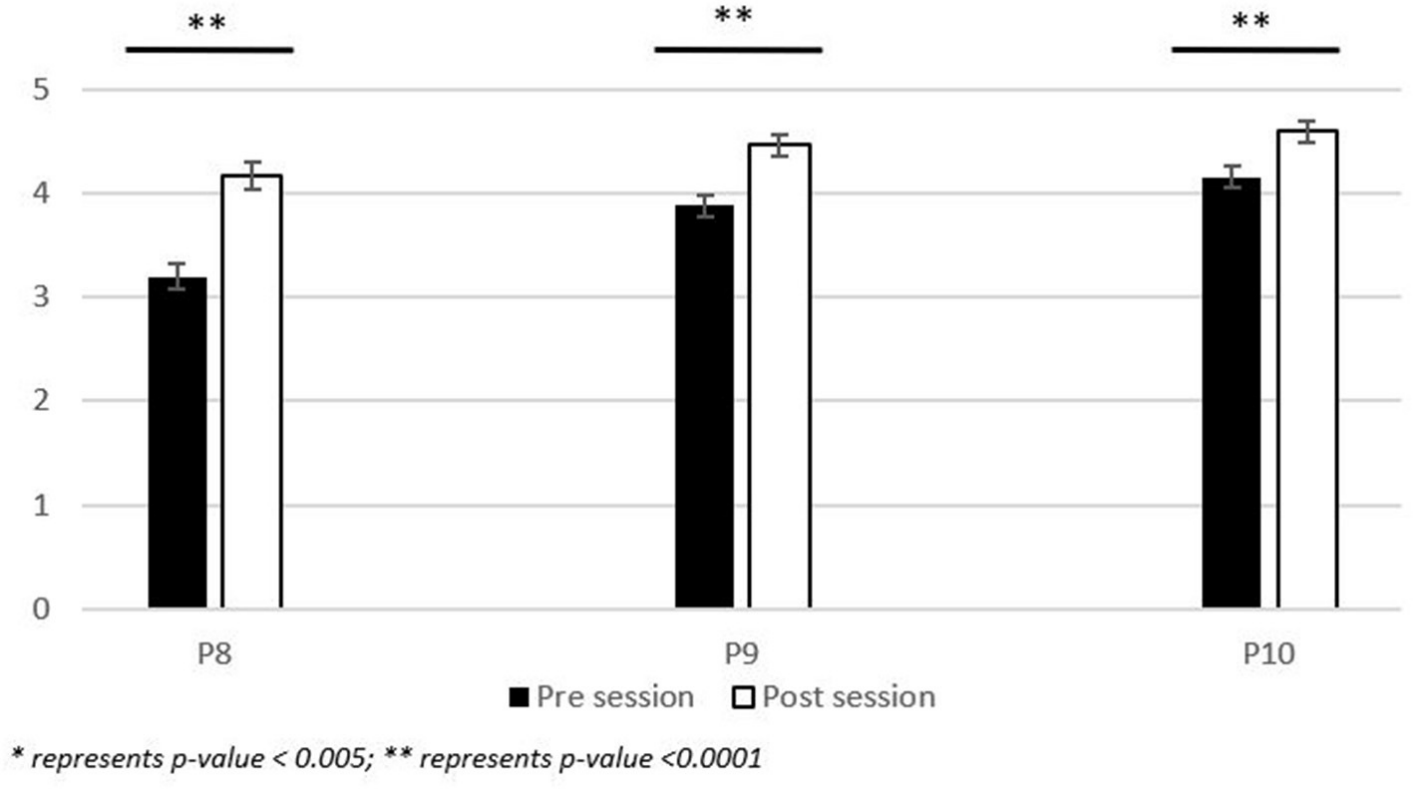
Participant reported change in the level of confidence related to LGBTQIA+ areas of concern. Range of significance of results of the pre-/post- activity survey responses (*n* = 111) to the following prompts: (P8) How confident are you in your knowledge of primary care of Lesbian, Gay Bisexual, and Transgender (LGBTQIA+) patients? (P9) How confident are you in your ability to identify implicit bias toward LGBTQIA+ individuals demonstrated by a colleague/classmate? (P10) How confident are you in your ability to create an environment which fosters others to comfortably disclose their gender identity to you? Participants responded on a 5-point Likert scale with 5, Extremely Confident; 4, Somewhat Confident; 3, Neutral; 2, Minimally Confident; 1, Not Confident at all. Data is represented as the average response from the Likert scale +/– the standard error of the mean.

Prompts 8 through 10 (P8 through P10) capture the student's level of confidence with LGBTQIA+ related areas of concern. Data is represented as a mean (Pre-activity vs. Post-activity data) +/– the standard error of the mean with a *p-*value of <0.05 considered as significant. From prompts P8 through P10: P8 (3.20 vs. 4.17; +/– 0.13), P9 (3.88 vs. 4.46; +/– 0.10), and P10 (4.15 vs. 4.59; +/– 0.10) were significant (*p-*value <0.001) (Figure 3).

### Key themes post session

**Figure 4:**
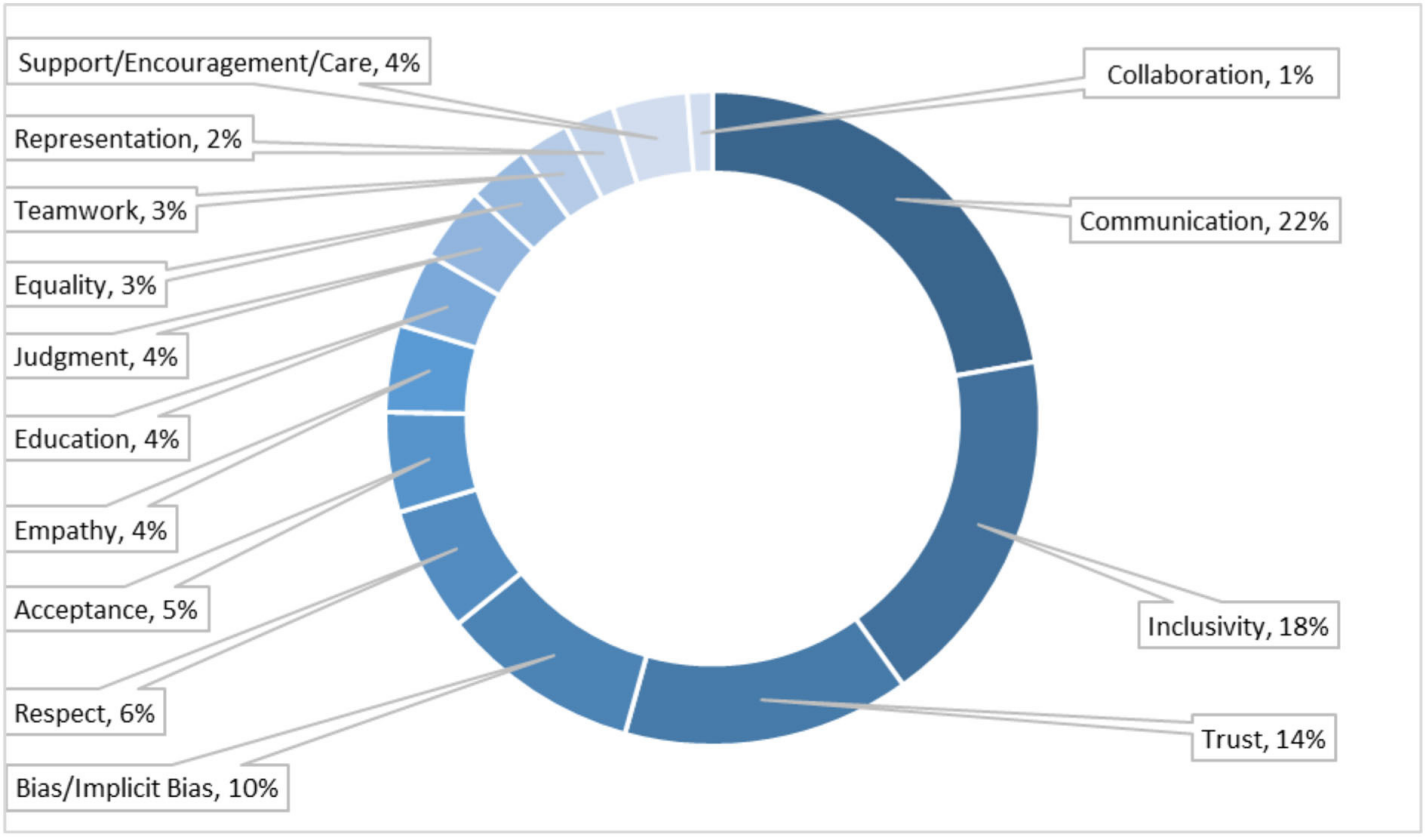
Key themes established through the session. Representation of common qualitative responses of the post-activity survey (*n* = 162, input responses) to the following prompt: P11: List any three Key Words/Phrases which come to your mind after this IPE activity? Participants responded with free-text input responses. The frequency of identical/ similar meaning words as a percent of total input responses. *Words with frequency of <2 are not shown.

From Prompt 11 (P11: List any three Key Words/Phrases which come to your mind after this IPE activity?), the prominent key themes that arose were Communication (22.22%), Inclusivity (17.90%), Trust (14.20%), Bias/Implicit Bias (9.88%), Respect (6.17%), Acceptance (4.94%), Empathy (4.32%), Education (3.7%), Judgement (3.7%), Equality (3.09%), Teamwork (2.47%), Representation (2.47%), Collaboration (1.23%) and Support, Encouragement, Care combined (3.7%) (Figure 4). This qualitative data is representative of the 162 entries entered in the post-activity survey for P11.

## Discussion

In 2021 and 2022, NSU hosted annual IPE Days that connected eight campuses and twenty professions, with an estimate of >1,300 students, using a synchronous online platform. The virtual setting provided an opportunity for the participation of multiple and diverse programs and campuses. Traditionally IPE activities can be seen as cumbersome (22, 23). This may be due to the involvement of multiple health professional programs and the logistics involved such as collaboration with programs and coordination for more participation. Based on our experience conducting our IPE activities it was evident that use of the virtual environment helped to overcome some of these obstacles and particularly, this session is transferable to any institution.

Participants' demographics showed a relatively equal distribution between colleges based on cohort size for each program and taking into account that the College of Healthcare Sciences offers ~10 distinct healthcare professional programs. However, there was a larger population of female (70.3%) participants in the study. Successful implementation of this early intervention through interprofessional education was evidenced by having 86.5% of student participants within their first 2 years of study in their programs (Table 1).

Limitations to the study include the absence of control over the group demographics, which is determined by the IPE Day administrators. This can be overcome in future sessions by pre-assigning the groups with an equal number of representations from each demographic category. Another factor that can have a considerable impact on the effectiveness of the session is the virtual setting of the discussion platform. In this study student engagement and interaction were high during the sessions however, improved efficiency of facilitators in the virtual setting would further enhance an environment conducive to student-driven learning.

Post-session, there was a significant (*p* < 0.005) increase in the student perspective on the importance of receiving education about primary care for LGBTQIA+ individuals and implementing gender-neutral care/procedures in clinical practice. Students also recognized the importance of engaging in self-reflection processes to address implicit biases regarding LGBTQIA+ individuals. This emphasis on self-reflection indicates support for the development of gender-neutral care/procedures in healthcare and being receptive to subsequent education and awareness.

Due to stakeholders in this experience being from various health professions programs at various stages of their education, the CBL was designed to be beneficial regardless of formal training directed toward the objectives of this session. Studies have shown that CBL can be successfully utilized early with students who have never participated in CBL before. Benefits of CBL early in the students' academic careers include providing context, experience using analytical reasoning, and the promotion of active student participation (24). Studies identify that CBL provides “deeper learning” that instead of the focus being that the learner identifies the correct answer, it “is more aligned with either evidence of critical thinking or changes in behavior and generalizability of learning to new cases” (17). The development of critical thinking along with four professional attributes of nursing students was positively influenced by CBL: (i) Salience of clinical knowledge; (ii) Multiple ways of thinking; (iii) Professional self-concept; and (iv) Professional attribute of caring (25, 26).

This session was successful in significantly enhancing the confidence (*p* < 0.001) of students in their knowledge of primary care of LGBTQIA+ patients and their ability to create a safe and inclusive environment. Students showed a significant change in their ability to identify implicit bias toward LGBTQIA+ individuals demonstrated by their colleagues and classmates. It may be concluded that only an hour-long activity can have a significant impact on the student interest and understanding of key challenges faced by LGBTQIA+ individuals. It is recommended that such opportunities continue with more frequency throughout the healthcare professional curriculums.

As presented in Figure 4, results obtained from the participant input section display words and phrases that align with the IPEC core competency of values/ethics such as “Equality”, “Judgment”, “Empathy”, “Support/Encouragement/Care”, etc. Other competencies such as interprofessional practice and communication were aligned with participant inputs such as “Collaboration” and “Communication” (15, 16). “Teamwork” included inputs that included teams and teamwork (15, 16). The inputs such as “Inclusivity”, “Trust”, “Bias/ Implicit-Bias”, “Representation”, “Respect”, and “Acceptance” express an appreciation for the LGBTQIA+ focus of the session. Overall participant responses indicate an emphasis on trust and patient care irrespective of the patient's sexual identity.

Students that engaged in this experience did not show a significant increase in the importance for primary care providers to be able to provide information to LGBTQIA+ patients about local resources for community engagement. This may be due to the emphasis of the session not being on community engagement, though it was discussed. In future iterations of the session, more emphasis could be placed on this aspect. For prompts related to the importance of recognizing the increased health risks and discussing safe sex practices associated with sexual orientation, the gap in knowledge was not as distinct between the pre-/post-session survey responses. As healthcare professional students, this was an expected outcome.

These results taken together indicate that the session objectives were fulfilled and received well by the students. This also represents that there is a need for more opportunities for training/sessions of this nature in the health professions to inculcate collaboration and standardized care for vulnerable groups such as the LGBTQIA+ community. This study yielded similar outcomes to those of Leslie et al.'s (27) study which demonstrated an increase in knowledge and in readiness for interprofessional education. One key difference in these studies is that the student population of the Leslie et al. study was from an institution that had laid a strong foundation of LGBT Health programming which contained 50 h of content related to LGBT healthcare, whereas this interactive session was for a population of students (at least 81%) who had limited or no previous exposure to content related to LGBT healthcare as shown in Figure 1 (27). McCave et al. (28) demonstrated that students displayed a need for additional training from their study employing IPE for LGBTQIA+ related topics. The study utilized transgender standardized patients for an IPE activity with students from Occupational Therapy (OT), Physical Therapy (PT), Medical Sciences, Physician Assistant (PA), Doctor of Medicine (MD), Social Work, Healthcare Administration, etc. healthcare programs (28). However, despite a positive impact on the students, there are only a limited number of published studies in this area, and more needs to be done to substantiate the intended widespread curricular change (27–29). With continued efforts in this field, expanded culturally competent interprofessional collaboration could be beneficial to improving healthcare for LGBTQIA+ patients. The IPE training network fosters simultaneous multifaceted delivery of appropriate training for numerous healthcare professions.

## Data availability statement

The raw data supporting the conclusions of this article will be made available by the authors, without undue reservation.

## Ethics statement

The studies involving human participants were reviewed and approved by Nova Southeastern University Institutional Review Board (IRB# 2021-12-NSU). The patients/participants provided their written informed consent to participate in this study.

## Author contributions

SP: conceptualized the idea, developed and facilitated the case and experience session, formulated data instruments, performed data analysis, and manuscript preparation. CO'M: developed the case, formulated data instruments, performed data analysis, and manuscript preparation. RD: developed and facilitated the case and experience session. AL: formulated data instruments and manuscript preparation. DG: developed and facilitated the case and experience session, formulated data instruments, and manuscript preparation. All authors contributed to the article and approved the submitted version.
