# Supplementary material for: Inclusive LGBTQIA+ healthcare: An interprofessional case-based experience for cultural competency awareness
Source: Front Public Health. 2023 Jan 4;10:993461. doi: 10.3389/fpubh.2022.993461 (PMC9846843; doi:10.3389/fpubh.2022.993461)
Supplement: Supplementary file 1 [file Data_Sheet_1.PDF]

Raven's Dilemma – An Interactive Case Based Discussion

Case Authors:
Samiksha Prasad, Ph.D.[1], **Chasity B O'Malley**, Ph.D.[1], **Rolando DeLeon**, M.D., F.A.C.O.G.[2], **Arkene S Levy**, Ph.D.[1], **Daniel P Griffin**, Ph.D.[1]

[1]Department of Medical Education, Dr. Kiran C Patel College of Allopathic Medicine, Nova Southeastern University
[2]HCA Mercy Hospital, Miami, FL, USA

Educational Objectives:
By the end of this session, learners will be able to:
1. Demonstrate being receptive to opinions of members of an interprofessional team in a patient-centered fashion. (IPEC domain: Communications)
2. Discuss and clarify each profession's scope of practice and the roles of each healthcare professions team member. (IPEC domains: Roles/Responsibilities and Communications)
3. Communicate the importance of teamwork in providing unbiased and inclusive patient-centered care. (IPEC domains: Teams/Teamwork and Ethics)
4. Recognize boundaries experienced by a marginalized patient population (IPEC domains: Ethics)

Part I:

Patient:
Raven is 24 years old and is presenting to a gynecology clinic with acute lower abdominal pain for one day. She identifies as Latinx and bisexual, but her current partner Lisa was not able to join her due to the COVID-19 restrictions in place.

Waiting Room:
She is in the waiting room for her OB/GYN appointment and is looking through informational pamphlets. She is disappointed to see few people of color on the pamphlets, and all couples portrayed as heterosexual. One of the fellow patients at the waiting room starts a conversation with her asking "Is your husband also waiting in the car like mine? It's unfortunate that due to COVID-19 they are not allowed to accompany us!". She smiles politely and only mentions that she drove there herself.

Prompt Questions:
- Why is Raven disappointed?
- How can the OB/GYN office change to make all patients feel included by the environment and other patients?
- What are the other things the practice can do to make it more welcoming for LGBTQI+ people?

Part II:

Nurse's Station:

Raven's name is called out and she is asked to fill out the intake form.
For the intake form the nurse proceeds to take the medical history. These include questions on contraception(s), children, last menstrual period (LMP) and sexual activity.
While inputting the information on the intake form, the nurse asks, "sexually active with men or women?"
When Raven says "Both", the facial expression of the nurse indicates surprise, which makes Raven feel uncomfortable.

Prompt Questions:

- What is the significance of the non-verbal communication expressed by the nurse?
- Does this affect Raven's openness in answering further questions by the nurse?
- How will Raven's experiences so far for this appointment predispose her interaction with the physician?
- How can the clinic personnel better communicate for better patient care?

Part III:

Exam Room:

The nurse instructs Raven to enter the room; completely disrobe; and put on a paper gown and lay down on the vinyl covered exam bed. She is told the doctor will be with her momentarily.
After 25 minutes pass, the doctor enters the room looking only at his laptop. He quickly begins to run through her chief complaint and history, before asking if she is on any type of birth control. The doctor stresses to her that since she is of reproductive age and not married, she should be on some sort of contraceptive method. Raven explains that she is in a monogamous same sex relationship and does not need contraception at this time.
The physician comments "But you also have intercourse with men, right?". Raven does not acknowledge the question and looks toward the door.
At this point, Raven is considering ending the appointment and leaving the clinic. She is still, however, in significant pain and wants to know what is causing it.
The physician proceeds with the examination, after which he performs an ultrasound which reveals a mid-cycle- follicular-ruptured ovarian cyst. After discussing the findings with Raven, he prescribes oral contraceptives but does not clearly explain that they are not primarily for contraception but rather to inhibit her ovulation, so she does not continue to form ovarian cysts.
Raven completes her appointment but due to her entire experience throughout the visit, she chooses not to fill the prescription and will just "see what happens".

Prompt Questions:

- How could have the physician perceived that Raven was uncomfortable and the visit was not proceeding in her best interest?
- How could the physician have made Raven feel more comfortable during her visit?
- How could the physician have educated Raven for the need for contraceptive for her case?

Part IV: the next month…

<u>At the Pharmacy</u>:

Raven feels intense pelvic pain again, thus she reluctantly decides to go to the local pharmacy for a walk-in consult. Lisa holds Raven's hand for support while they are waiting at the booth for the Pharmacist. When the pharmacist on site arrives, he asks very politely: "Is it ok if I discuss your medication plan in front of your partner?"

Raven agrees and explains to him that she did have the same pain a month ago and went to the doctor's office for it but only received a prescription for contraception medication from there.

The Pharmacist was very polite and empathetic and explains to Raven that the birth control medication will help in avoiding ovulation, and that in turn will prevent further ovarian cysts from forming.

Now she feels much more comfortable in starting to take the medication.

Prompt Questions:

- What did the pharmacist do to help Raven with her situation?
- How was the pharmacist's interaction with Raven and Lisa?

Part 5: A few months later...

<u>Dentist Appointment</u>:

Raven was apprehensive about her mid-year check up at the Dental clinic after how her last appointment went at the doctor's office. However, Lisa recommends Raven go to the dentist Lisa has been going to. Raven decides to give it a try.

Immediately upon entering the office, she is glad to see educational posters which depicted heterosexual as well as same sex couples.

The intake form included many descriptors and a "prefer not to answer" option for sexual orientation and identity. This made Raven feel comfortable.

The dentist welcomes Raven with a very pleasant smile and asks how she learned about their clinic. Raven explains her partner Lisa introduced her to this dental clinic. The dentist appreciates and notes that Raven has a very supportive partner.

During the procedure, Raven feels very comfortable and accepted.

The appointment goes well, and Raven feels much more comfortable with the interaction she had at this healthcare facility and is much more encouraged to keep up with her future appointments.

Prompt Questions:

- How was this interaction a more positive experience for Raven?
- What can you as a health professional do to help create more positive experiences like this?
- Now that we are increasingly aware of the problem, how can we fix this? *(Consider thinking from an interprofessional approach to care)*
