# Supplementary material for: Inclusive LGBTQIA+ healthcare: An interprofessional case-based experience for cultural competency awareness
Source: Front Public Health. 2023 Jan 4;10:993461. doi: 10.3389/fpubh.2022.993461 (PMC9846843; doi:10.3389/fpubh.2022.993461)
Supplement: Supplementary file 2 [file Data_Sheet_2.pdf]

Appendix 2_ Data Instrument

1. Which HPD college are you associated with? __________

2. Which Program are you enrolled in? ____________

3. What year of study are you in for your program?
   - Year 1
   - Year 2
   - Year 3
   - Year 4

4. What is your age?

   - under 20
   - 20 - 25
   - 26 - 30
   - 31 - 35
   - 36 - 40
   - 41 - 45
   - 46 - 50
   - 51 or higher
   - Prefer not to answer

5. What is your gender? _______________

6. What is your anticipated career path? ______________

7. How much prior teaching have you received in your current program exclusive to the care of lesbian, gay, bisexual, and transgender (LGBTQI+) patients?
   - Less than 1 hour
   - 1 - 2 hour(s)
   - 3 - 4 hours
   - More than 5 hours
   - None

8. How important is it for healthcare professional students to receive education about the primary care of Lesbian, Gay and Bisexual, and Transgender patients?
   - Extremely important
   - Somewhat important
   - Neutral

- Somewhat unimportant
- Extremely unimportant

9. How important is it for healthcare professional students to receive education about the primary care of Transgender patients?
   - Extremely important
   - Somewhat important
   - Neutral
   - Somewhat unimportant
   - Extremely unimportant

10. How important is it for a primary care provider to be able to provide information to (LGBTQI+) patients about local resources for (LGBTQI+) community engagement?
    - Extremely important
    - Somewhat important
    - Neutral
    - Somewhat unimportant
    - Extremely unimportant

11. How important is it for healthcare professional students to recognize increased health risks associated with sexual orientation?
    - Extremely important
    - Somewhat important
    - Neutral
    - Somewhat unimportant
    - Extremely unimportant

12. How important is it to engage in self-reflection processes to correct implicit biases regarding LGBTQI+ individuals?
    - Extremely important
    - Somewhat important
    - Neutral
    - Somewhat unimportant
    - Extremely unimportant

13. How important is it is to implement gender-neutral practices in your clinical practice and clinic?
    - Extremely important
    - Somewhat important
    - Neutral
    - Somewhat unimportant
    - Extremely unimportant

14. How important is it to discuss safe sex practices with individual women who have sex with women?

- Extremely important
- Somewhat important
- Neutral
- Somewhat unimportant
- Extremely unimportant

15. How confident are you in your knowledge of primary care of Lesbian, Gay Bisexual, and Transgender (LGBTQI+) patients?
    - Extremely confident
    - Somewhat confident
    - Neutral
    - Minimally confident
    - Not confident at all

16. How confident are you in your ability to identify implicit bias towards LGBTQI+ individuals demonstrated by a colleague/classmate?
    - Extremely confident
    - Somewhat confident
    - Neutral
    - Minimally confident
    - Not confident at all

17. How confident are you in your ability to create an environment which fosters others to comfortably disclose their gender identity to you?
    - Extremely confident
    - Somewhat confident
    - Neutral
    - Minimally confident
    - Not confident at all

The following questions were included only in the post survey:

18. List any three Key Words/Phrases which come to your mind after this IPE activity?

    ____

19. The written materials for this case were informative.
    - Strongly agree
    - Agree
    - Neutral
    - Disagree
    - Strongly disagree

20. From this case which TWO were the most important or impactful to your education. (Check all that apply)
    - Understanding LGBTQI+ issues
    - Cultural competency; performing a sensitive history and physical
    - Health promotion and disease prevention
    - Mental health and societal factors

21. Reflecting back on the education and training you have received in your program so far; do you feel the time spent on this topic (i.e. Optimal Care for LGBTQI+ patients) was ______________.
    - not invested at all
    - too short
    - just right
    - too much time

22. The curriculum AS A WHOLE increased my understanding of the barriers faced by patients who identify as LGBTQI+ when interacting with the healthcare system.
    - Strongly disagree
    - Disagree
    - Neutral
    - Agree
    - Strongly agree
